# Targeting Telomerase and ATRX/DAXX Inducing Tumor Senescence and Apoptosis in the Malignant Glioma

**DOI:** 10.3390/ijms20010200

**Published:** 2019-01-08

**Authors:** Hueng-Chuen Fan, Chuan-Mu Chen, Ching-Shiang Chi, Jeng-Dau Tsai, Kuo-Liang Chiang, Yu-Kang Chang, Shinn-Zong Lin, Horng-Jyh Harn

**Affiliations:** 1 Department of Pediatrics, Department of Medical research, Tungs’ Taichung Metroharbor Hospital, Wuchi, Taichung 435, Taiwan; fanhuengchuen@yahoo.com.tw (H.-C.F.); chi-cs@hotmail.com (C.-S.C.); yogurt8306@gmail.com (Y.-K.C.); 2 Department of Life Sciences, National Chung Hsing University, Taichung 402, Taiwan; chchen1@dragon.nchu.edu.tw; 3 Department of Rehabilitation, Jen-Teh Junior College of Medicine, Nursing and Management, Miaoli 356, Taiwan; 4 The iEGG and Animal Biotechnology Center, and Ph.D. Program in Translational Medicine, National Chung Hsing University, Taichung 402, Taiwan; 5 School of Medicine, Chung Shan Medical University, Taichung 402, Taiwan; fernand.tsai@msa.hinet.net; 6 Department of Pediatrics, Chung Shan Medical University Hospital, Taichung 402, Taiwan; 7 Department of Pediatric Neurology, Kuang-Tien General Hospital, Taichung 433, Taiwan; lambier.tw@yahoo.com.tw; 8 Department of Nutrition, Hungkuang University, Taichung 433, Taiwan; 9 Buddhist Tzu Chi Bioinnovation Center, Tzu Chi Foundation, Hualien 970, Taiwan; shinnzong@yahoo.com.tw; 10 Department of Neurosurgery, Buddhist Tzu Chi General Hospital, Hualien 970, Taiwan; 11 Department of Pathology, Buddhist Tzu Chi General Hospital and Tzu Chi University, Hualien 970, Taiwan

**Keywords:** glioblastoma multiforme (GBM) 2, telomerase 3, alternative lengthening of telomeres 4, α-thalassemia/mental retardation syndrome X-linked protein (ATRX) 5, death-domain associated protein (DAXX) 5, butylidene phthalide (BP)

## Abstract

Glioblastoma multiforme (GBM) is a type of brain tumor that is notorious for its aggressiveness and invasiveness, and the complete removal of GBM is still not possible, even with advanced diagnostic strategies and extensive therapeutic plans. Its dismal prognosis and short survival time after diagnosis make it a crucial public health issue. Understanding the molecular mechanisms underlying GBM may inspire novel and effective treatments against this type of cancer. At a molecular level, almost all tumor cells exhibit telomerase activity (TA), which is a major means by which they achieve immortalization. Further studies show that promoter mutations are associated with increased TA and stable telomere length. Moreover, some tumors and immortalized cells maintain their telomeres with a telomerase-independent mechanism termed the “alternative lengthening of telomeres” (ALT), which relates to the mutations of the α-thalassemia/mental retardation syndrome X-linked protein (ATRX), the death-domain associated protein (DAXX) and H3.3. By means of the mutations of the telomerase reverse transcriptase (TERT) promoter and ATRX/DAXX, cancers can immortalize and escape cell senescence and apoptosis. In this article, we review the evidence for triggering GBM cell death by targeting telomerase and the ALT pathway, with an extra focus on a plant-derived compound, butylidene phthalide (BP), which may be a promising novel anticancer compound with good potential for clinical applications.

## 1. Introduction

Despite their low incidence rate (25.48 per 100,000 person-years) [1], brain tumors are one of the most frightening diseases, not only because of their high morbidity and mortality rate but also because of the heavy burden they place on patients, their loved ones, and healthcare systems. Glioma, accounting for nearly 80% of all malignant primary tumors of the brain, is classified into astrocytic tumors, including astrocytoma, anaplastic astrocytoma, and glioblastoma multiforme (GBM); oligodendrogliomas; ependymomas, and mixed gliomas, according to their presumed cell of origin [2]. The previous version of the World Health Organization Classification of Tumors of the Central Nervous system (CNS) had classified gliomas into grade I to IV. Grade I gliomas possess low proliferative potential; grades II to IV gliomas are highly invasive and malignant. GBM, a grade IV glioma, is the most aggressive, invasive, and undifferentiated type of tumor. The classification is based on the level of malignancy, which is weighed by the histopathological criteria [3]. However, numerous studies have discovered a significant interobserver variation in the histological diagnosis of gliomas that may affect the typing and grading of glial tumors and subsequently affect the dosages used in radiotherapy and chemotherapy [4]. Since treatment decisions and prognoses are based on histological diagnoses and grading, the latest version of the World Health Organization CNS tumors classification has undergone a complete transformation by incorporating molecular criteria in addition to the pre-existing histological parameters to minimize subjective interpretation of morphological descriptions, as well as interobserver variation, and to provide more objective, quantitative, and reproducible results for recording the grade and lineage of gliomas [5]. GBM, the most malignant type of glioma, accounts for approximately 20% of all brain tumors [6], and total age-adjusted incidence rates of GBM range from 0.59 to 3.69 per 100,000 persons in the world [7]. The incidence rates of GBM in the United States and Taiwan are 3.19 and 0.85 per 100,000 person-years, respectively [8,9]. GBM can occur at any age [10], but is rare in children, and commonly occurs in older patients (mean age = 64 years) [11]. Men have a higher incidence of GBM than women [12]. GBM is notorious for its high mortality rate because only 0.05 to 4.7% of patients survive five years past diagnosis [7]. Current treatment options for GBM include maximal surgical resection, followed by temozolomide and radiation [13]. However, a complete resection is always difficult and postsurgical treatment is usually necessary to prevent recurrence. At the same time, suboptimal penetration through the blood–brain barrier (BBB), drug toxicity, and drug resistance remain major obstacles to the success of chemotherapy [14]. In general, the prognosis of GBM is poor. Even with maximal surgical resection plus radiotherapy with concomitant or subsequent chemotherapy, overall, patients usually have a median survival of approximately 14–15 months from diagnosis [12,15] and the median survival for untreated patients is only three months [16]. The diffuse, invasive nature and location of brain tumors suggest that it is barely possible for an effective treatment to destroy all tumor cells. This reality underscores the need for continuing investigations of novel and alternative therapeutic options, including clinical trials of any agents showing therapeutic potential.

## 2. Clinical Characteristics of GBM

**Figure 1:**
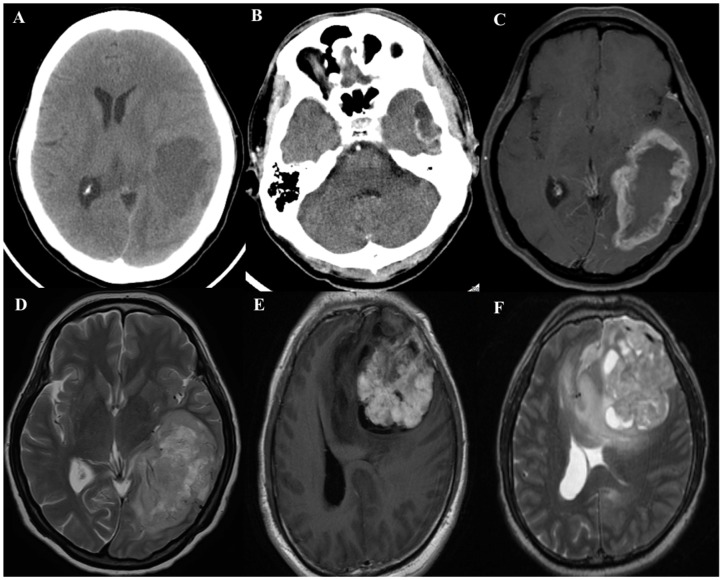
GBM. (**A**) The axial brain CT without contrast shows an irregular mass with central necrosis over the left temporo-parieto-occipital lobes with prominent edematous effect with midline-shifting. (**B**) The axial brain CT with contrast shows multiple lobulated cystic structures with enhanced wall and septi over the left temporal lobe. (**C**) The axial brain magnetic brain imaging, T1-weighted repetition time (TR), 450 ms; echo time (TE), 10 s, Achieva 1.5 T, Philips] image with gadolinium contrast demonstrated an irregular rim enhanced mass with large central necrosis at the left temporo-parieto-occipital lobes. The prominent edematous effect completely compressed the occipital horn of the left lateral ventricle. (**D**) The axial brain MRI, T2-weighted (TR, 4980 ms; TE, 100 s) image with gadolinium contrast demonstrated an irregular ovoid mass with necrosis at the left temporo-parieto-occipital lobes. The edematous effect and mass effect were prominent. (**E**) The axial brain MRI, T1-weighted image with gadolinium contrast demonstrated an irregularly enhanced mass with central necrosis involving the left frontal lobe. The prominent edematous change resulted in brain herniation across the midline. (**F**) The axial T2-weighted image showed a remarkable perifocal edema in the left frontal lobe, extending to the insula, and corpus callosum genu with subfalcine herniation.

Patients with brain tumors may present with various signs and symptoms, such as severe, persistent, or recurrent headache; nausea and vomiting; papilledema; seizures; focal neurological and cognitive impairments, such as difficulty in speaking or thinking of words; disturbed vision, hearing, smell, or taste; weakness or paralysis in part of the body; loss of balance; general irritability; drowsiness or a change in personality; flashbacks; and loss of memory [17]. In general, the clinical history of patients with a low-grade astrocytoma may span several years. GBM may present with a short 3–6-month clinical history. Occasionally, the symptoms may develop rapidly, which might be mistaken for a stroke [2]. Moreover, 95% of GBM cases emerge in the supratentorial region, but they can occur in all cortical areas, the brainstem, and the spinal cord [18]. In general, the neuroimaging of GBM is not specific. Some cases may show an irregular lesion with central low-density (Figure 1A) or multiple cystic structures with an enhanced wall on computed tomography (CT) (Figure 1B) or irregular rim appearance with central necrosis (Figure 1C), or a multi-lobular mass and prominent peritumoral edema with distortive structures of the surrounding brain and ventricles on magnetic resonance imaging (MRI) (Figure 1D–F). The differential diagnoses of the imaging findings are abscess, metastasis, lymphoma, multiple sclerosis, and subacute infarctions [18]. The typical outlook of GBM, which is a single, large, irregular lesion in the white matter, consists of necrosis, cystic and gelatinous areas, and multifocal hemorrhage, causing intermixing of firm and soft textures [19]. Histologically, GBM shows variable cells, from small poorly differentiated cancer cells to large multinucleate cancer cells with multifocal necrosis with pseudopalisading nuclei and high mitotic activity, and proliferative vascular endothelial cells with a glomeruloid structure [19].

## 3. Risk Factors

In the past, only a few GBM risk factors were identified as being linked to environmental stimuli, including poor diet, smoking, cellular phones usage or exposure to an electromagnetic field, severe head injury, occupational risk factors and pesticide exposure [19,20,21,22,23]. Steroid hormones were suspected as a possible cause for GBM [24]. Exposure to high-dose radiation is the only confirmed risk factor in extensive retrospective cohort data [23,25]. Allergic conditions, including asthma, hay fever, eczema, and food allergies, may have a protective effect against GBM [26,27,28,29], and a meta-analysis study has demonstrated the risk of developing gliomas reduces to 40% in people who have allergies [30], suggesting that immunosurveillance may inhibit the growth of a glioma. Several diseases such as neurofibromatosis type 1 and type 2 [31], tuberous sclerosis (TSC1 and TSC2) [32], Lynch syndrome [33], melanoma-astrocytoma syndrome [34], Ollier disease/Maffucci syndrome [35], and Li-Fraumeni syndrome [36], are associated with the risk of developing gliomas, but only 5–10% of cases of gliomas are reported to show genetic predisposition [21]. The results indicate that the polygenic, instead of the monogenic, model, may explain the incidence pattern of adult gliomas, and results from genome-wide association studies have supported this conclusion by identifying variations in eight genomic regions as contributing to the risk of developing gliomas: telomerase RNA component (*TERC*), telomerase reverse transcriptase (*TERT*), epidermal growth factor receptor (*EGFR*), coiled-coil domain containing 26,cyclin-dependent kinase inhibitor 2B, pleckstrin homology such as domain family B member 1, tumor protein *p53* *(TP53)*, and the regulator of telomere elongation helicase 1 (RTEL1) [37,38,39,40,41]. Three of these glioma risk loci (*TERC*, *TERT*, and *RTEL1*) contain genes involved in telomere maintenance. Moreover, recent publications demonstrate that acquired somatic mutations in *TERT* and α-thalassemia/mental retardation syndrome X-linked protein (*ATRX*) can affect telomere maintenance in tumor cells and are important in glioma development and prognosis [42,43,44,45]. The present review focuses on novel and alternative therapeutic options for treating GBM by inducing tumor senescence and apoptosis through the mechanisms of telomerase and ATRX/death-domain associated protein (DAXX).

## 4. Etiology and Pathogenesis of GBM

The etiology of GBM is unknown. GBM can be divided into primary and secondary GBMs, based on clinical or histological evidence. Primary GBMs develop rapidly de novo in older patients without clinical or histological evidence of less malignant precursor lesions. Secondary GBM develops more slowly, from low-grade diffuse astrocytoma or anaplastic astrocytoma, in young patients with a significantly better prognosis. Histologically, primary and secondary GBMs are similar, but they differ in their genetic profiles, including the mutations of isocitrate dehydrogenase (IDH), the co-deletion of chromosomes 1p and 19q, mutations of H3F3A, telomerase, TERT, and ATRX.

### 4.1. Isocitrate Dehydrogenase (IDH)

To elucidate genetic alterations in GBM patients with varying prognoses or responses to specific targeted therapies and to identify subgroups of GBM patients for a better histopathological classification, an integrated genomic analysis was used to identify mutations inisocitrate dehydrogenase 1(*IDH1*) in 12% of patients with GBM [46]. It was subsequently reported that GBMs without *IDH1* mutations often have mutations of isocitrate dehydrogenase 2 (*IDH2*) [47]. The structures of IDH1 (located on chromosome 2q33.3) and IDH2 (located on chromosome 15q26.1) are homodimeric and share similar sequences and an almost identical protein structure [48]. IDH1 and IDH2 encode two separate, different isocitrate dehydrogenase enzymes, which are nicotinamide adenine dinucleotide phosphate (NADP^+^)-dependent, catalyze oxidative decarboxylation of isocitrate to α-ketoglutarate (α-KG), and reduce oxidized nicotinamide adenine dinucleotide (NAD^+^) or NADP^+^ to NADH or NADPH. IDH1, localizing in the cytoplasm and peroxisomes, involves cellular metabolism and protection from reactive oxygen species and radiation [49,50]. IDH2, localizing in the mitochondria, is associated with the regulation of the tricarboxylic acid cycle and protection from oxidative stress [50]. *DH1* and *IDH2* mutations are mono-allelic, somatic, and missense changes. Mutations in *IDH1* commonly affect R132, which is the binding site for isocitrate [46]. Mutations in *IDH2* only affect R172 and R140 [47,51]. Mutant IDH possesses catalytic activity to convert α-KG to 2-hydroxyglutarate (2-HG) [52]. Excessive 2-HG is a metabolic hallmark of certain subtypes of gliomas [53]. Both mutated IDH1 and IDH2 are common in adult gliomas (WHO grades II and III) and secondary GBM (WHO grade IV). Mutated *IDH1* and *IDH2* are very rare in childhood GBM [54], suggesting gliomas with mutated IDH are clinically and genetically different from those with wild-type (WT) IDH genes.

### 4.2. Co-Deletion at Chromosome Regions 1p/19q

The complete deletion of chromosomes 1p and 19q is common in oligodendrogliomas and occur in 50–70% of both low-grade and anaplastic tumors [55,56,57,58]. These findings suggest that chromosomes 1p and 19q may contain tumor suppressor genes, including the far upstream element binding protein 1 (*FUBP1*) on chromosome 1p and the capicua transcriptional repressor (*CIC*) on chromosome 19q [59,60,61]. The *FUBP1* expresses a single-stranded DNA-binding protein that can bind to several DNA regions, which harbor the far upstream element (FUSE) that is localized in the upstream of c-Myc. One function of FUSE is to regulate c-Myc in undifferentiated cells [62]. CIC, a tissue-specific transcriptional repressor, is expressed in the developing CNS and its dysfunction is associated with spinocerebellar ataxia type 1. This CIC-DNA interaction can be inhibited through the activation of the receptor tyrosine kinase (RTK) core signaling molecule mitogen-activated protein kinase (MAPK), which then allows for the transcription of CIC targets through this RTK-MAPK signaling axis. CIC alterations are common in specific cancer types (e.g., oligodendroglioma and Ewing-like sarcomas) [63]. Two clinical trials have clarified associations between combined 1p/19q co-deletion and an improved chemotherapeutic response and prognosis in oligodendrogliomas [64,65]. However, partial 1p or 19q deletion is more common in astrocytic tumors and secondary GBM but rare in primary GBM [66,67,68].

### 4.3. Mutations of H3F3A

Inside the nucleus, DNA, RNA and proteins form chromatin, which packs the DNA to a smaller volume and prevents the long DNA strands from being tangled. The structure of the chromatin is like “beads on a string”. The nucleosome is the “bead”, which is a basic unit of chromatin. Histones, nuclear proteins, can store DNA, modulate chromatin structure, impact gene expression, and regulate almost all DNA metabolic processes through post-translational modification, which includes methylation, phosphorylation, acetylation, ubiquitylation, and sumoylation [69]. Each nucleosome is composed of a histone octamer, which is composed of two copies each of the core histone H2A, H2B, H3, and H4. H1, which does not contribute to the nucleosome bead, binds to the linker DNA region between nucleosomes, helping pack the chromatin into higher order structures. A chain of nucleosomes is compacted to form a chromosome [70]. Histone H3s, which have several variants, include H3.1, H3.2, and H3.3. H3.1 is encoded by HIST1H3A-J.H3.2 is encoded by HIST2H3A, HIST2H3C, and HIST2H3D. H3.3 is encoded by H3F3A and H3F3B [71]. Numerous H3 lysine residues can be post-translationally modified, including acetylated at lysines 9, 14, 18, 23, and 56; methylated at arginine 2 and lysines 4, 9, 27, 36, and 79, and phosphorylated at ser10, ser28, Thr3, and Thr11 [72]. Mutation of H3F3A, including K27M substitution, in which a lysine residue on the histone H3 tail is substituted for a methionine, were discovered in diffused intrinsic pontine glioma(DIPG) [73]. As the location of the *H3F3A* at *lysine 27* is at or near critical regulatory histone residues, therefore, the alternations of mutant K27M on genes, which should be silent, produce widespread aberrant DNA methylation and deregulation of gene expression, impede physiological differentiation and to drive cell transformation [74]. Furthermore, in the WHO CNS tumor classification 2016, the principle of an integrated diagnosis was introduced with the combination of histological and molecular features, exemplified in the novel entity “diffuse midline glioma, H3K27M- mutant” [5]. The mutation in HIST1H3B-C, have been detected in approximately 10% of DIPG [75]. Another mutation of H3F3A, encoding a glycine 34 to arginine or valine (G34R/V) substitution, is reported in a smaller portion of pediatric and young adult high-grade astrocytoma [54]. H3F3G34 mutations may drive pediatric GBM through mismatch repair (MMR) deficiency [76] and upregulation of v-myc myelocytomatosis viral related oncogene, neuroblastoma derived (avian) (MYCN) [77].

### 4.4. Telomeres and Telomerase

**Figure 2:**
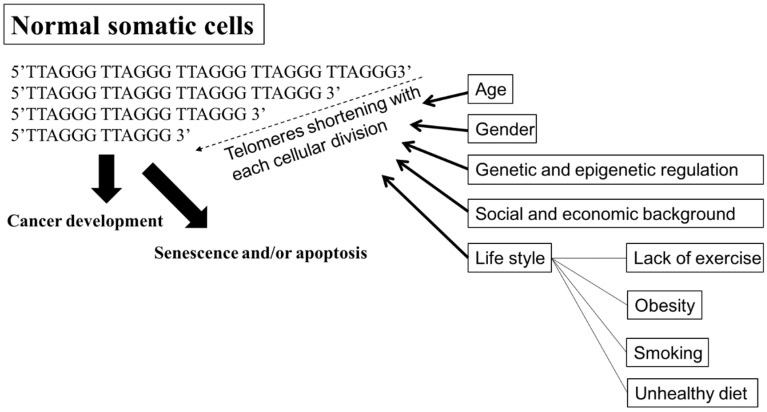
Factors affecting the rate of shortening telomeres. Telomeres are regions of repetitive nucleotide sequences, TTAGGG. Telomeres shorten in each cell division due to incomplete DNA replication. When telomere length progressively reduces to a critical point, the cell then executes senescence or apoptosis, or develops cancer. Several factors, including age; gender; genetic and epigenetic regulation; social and economic background; and life style, such as lack of exercise, obesity, smoking, and an unhealthy diet, are associated with increasing the rate of losing telomeres, leading to senescence or apoptosis, premature death, and cancer development. Bold arrow: shortening DNA leading to senescence and/or apoptosis, or cancer development. Arrow: factors affecting telomeres shortening.

A telomere is a capping structure located at the end of a linear chromosome. As most prokaryotes have circular chromosomes, they do not have telomeres; on the other hand, telomeres in vertebrates consist of a region of repeats of the six-nucleotide sequence TTAGGG at the ends of chromosomes, which themselves carry the complementary DNA strand sequence, AATCCC. In humans, the TTAGGG sequence repeats in tandem approximately 3000–20,000 times [78,79]. The sequence is bound by the shelterin complex, which is formed by TRF1, TRF2, TPP1, TIN2, POT1 and RAP1 [80]. Telomeres are known to maintain the stability of chromosomes and protect genes [81,82]. Chromosomes without this capping structure, meanwhile, become truncated and fuse with neighboring chromosomes [83]. In the process of chromosome replication, the synthesis of Okazaki fragments requires that RNA primers attach to the lagging strand. The shedding RNA then causes telomere shortening. As such, the accumulative loss of telomeres can eventually cause cell cycle arrest and apoptosis, leading to speculation that the progressive reduction in telomere length may play a key role in determining the timing of in vitro cellular senescence [84,85]. Therefore, telomeres are also viewed by some as a sort of biological clock that controls normal cell proliferation [86]. It is estimated that the human telomere loses approximately 24.8 to 27.7 base pairs per year [87,88]. There are several factors, however, that affect the rate of telomere shortening, including the host’s age [89]; gender [90]; genetic and epigenetic regulation [91,92,93]; social and economic background [94,95], and life style factors, such as the lack or presence of exercise, obesity, smoking, and unhealthy diets [88,95,96] (Figure 2). Individuals with shorter telomeres are known to be associated with various age-related diseases and conditions, such as heart failure [97], coronary heart disease [98,99,100], diabetes [101], osteoporosis [102], and a shorter lifespan [103,104,105]. Although the shorter length of telomeres is generally thought to be a marker of poor health and aging, it can lead to genomic instability [106,107,108] and elevated telomerase activity (TA) [109,110], resulting in a potential cancer predisposition factor.

Cell division leads to progressive telomere shortening, resulting in cell senescence; however, the shortening of telomeres can be counteracted by telomerase [111]. Telomerase, an RNA-dependent DNA polymerase, is expressed in developing embryos, in reproductive cells (i.e., proliferating germlines), in activated immune cells, and transiently, in adult stem cells, but telomerase is turned off in most adult human tissues. In immortalized cells, telomere length remains stable, with the activation of telomerase being considered one of the main mechanisms underlying this stability [111,112,113]. TA is exhibited in almost all human tumors and in tumor-derived cell lines, while most human somatic cells do not display TA, except for highly proliferative cells, such as bone marrow cells [114,115]. Telomerase, which is made up of TERT, TERC, and specialized proteins (e.g., dyskerin), preserves telomere stability by adding TTAGGG repeats to the end of the given chromosome (capping) in rapidly dividing cells [112,113,116], using its complementary TERC sequence as the template [117], together with TERT subunit as the catalytic component [111]. Activities of TERT are frequently up-regulated in human cancers, which is thought to be a critical mechanism contributing to human tumorigenesis [118,119]. Studies have identified two cancer-specific *TERT* promoter mutations (C228T and C250T) [44] in the activation of telomerase in various cancer cells [120,121], including GBM [122]. The two mutations in the *TERT*, which cause *TERT* activation to increase TA to elongate telomere length [44,123], lead to the proliferative, anti-senescence, and immortal properties of tumor cells. Mutations in the TERT promoter have been detected in more than 50% of primary adult GBM, and these mutations are correlated with EGFR, IDH1, IDH2, TP53, and ATX mutations and increased TA [45,123,124,125,126]. Mutations in the TERT promoter have been detected in 3–7% of pediatric GBM [44,123] and tumor cells in this group maintain or increase telomere length through alternative lengthening of telomeres (ALT) pathway [127,128].

### 4.5. Alternative Lengthening of Telomeres, α-Thalassemia X-Linkedintellectual Disability, and Death-Domain-Associated Protein

TA was detected in 76% of cervical cancer cases [129], 54% of medullary thyroid cancer cases [130], 42% of well-differentiated papillary thyroid cancer cases [131], 86.6% of non-small cell lung cancer cases [132], and > 80% of hepatocellular carcinoma cases [133]. Although telomerase reactivation is the most common mechanism of telomeric repeat addition in cancers [109], there is a convincing argument that TA is not the only way for tumors to become immortalized; otherwise, these tumors would have shrunk and died unless they managed to boost their TA to maintain their telomere length. Additionally, Indeed, 5–10% cancer cases exploit a telomerase-independent mechanism to elongate their telomeres, a phenomenon that is also known as ALT [134]. In addition to immortalized cells and cancer cells, ALT exists in non-neoplastic tissues, in endothelial, stromal, and some epithelial cells [135]. Although the prevalence of the ALT phenotype in cancers is low, ALT is common in certain cancer subtypes, including gliomas [136]. While primary GBM has been found to employ telomerase activation, nearly 75% of WHO grades II–III astrocytomas and secondary GBMs, with normal telomerase expression and WT TERT promoter, was observed to employ ALT for the maintenance of telomere length and genome stability [60,137]. The transformation from telomerase-dependent to ALT-mediated telomere lengthening is considered one of the strategies cancer cells adopt to escape cell senescence and apoptosis caused by telomerase dysfunction or absence [138,139]. ALT, which is not present in normal cells, often begins with a loss of chromatin remodeling proteins in the telomeres, with a resulting DNA damage response, recombination, and abnormal protein behavior that initiates ALT [140].

**Figure 3:**
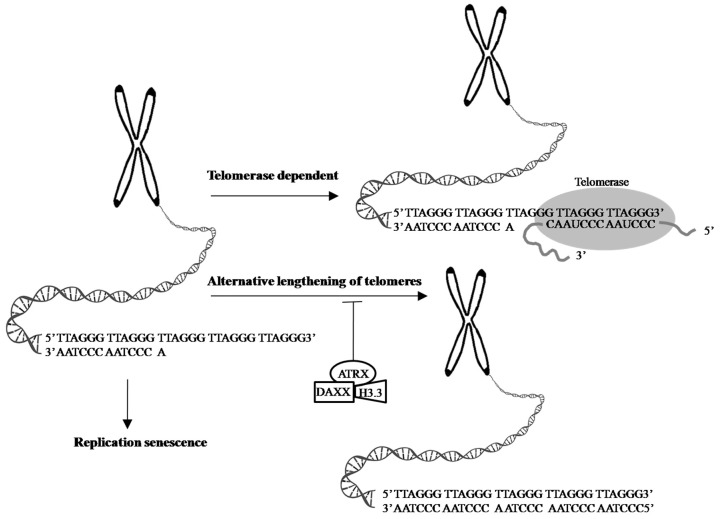
Telomeres are cap-like features at the ends of chromosomes that help protect them when cells divide. Telomeres contain thousands of repeats of the six-nucleotide sequence TTAGGG at the chromosome ends, with complementary DNA strand sequences AATCCC. 1. In the process of cell division, chromosome replication causes progressive telomere shortening, resulting in cell cycle arrest and apoptosis, leading to cellular senescence. 2. The shortening of telomeres can be counteracted by telomerase, which is made up of TERT, TERC, and specialized proteins. Telomerase maintains the length of telomeres stability by adding TTAGGG repeats to the end of the given chromosome, using its complementary TERC sequence as the template, together with TERT subunit as the catalytic component. 3. ALT is a phenomenon that 5–10% cancer cases exploit a telomerase-independent mechanism to elongate their telomeres [134]. The transformation from telomerase-dependent to ALT-mediated telomere lengthening may be one of strategies cancer cells adopt to escape cell senescence and apoptosis caused by telomerase dysfunction or absence [138,139]. ATRX, in collaboration with DAXX and H3.3 promotes the processes of telomere shortening [127,144,153]. Evidence shows the ATRX/DAXX/H3.3 complex in ALT suppression and a good prognostic factor in GBMs [143]. Arrow: possible pathway. T-bar: inhibition.

α-thalassemia X-linked intellectual disability (ATRX) syndrome is characterized by distinctive craniofacial features, genital anomalies, severe developmental delays, hypotonia, intellectual disability, and mild-to-moderate anemia secondary to α-thalassemia [141].The ATRX gene, which encodes a SWItch (SWI)/sucrose non-fermenting(SNF)-like chromatin remodeling protein, is frequently mutated in a variety of tumors, including adult lower-grade gliomas, pediatric GBM pediatric adrenocortical carcinoma, osteosarcoma, and neuroblastoma [142]. Cancer cells with a loss of ATRX gene display large and bright telomeric DNA foci that are significantly correlated with ALT [127], suggesting that ATRX may be a suppressor of the ALT mechanism and a good prognostic factor in cancers, such as in GBMs [143]. Moreover, the forced expression of ATRX in ALT-positive and ATRX-negative cell lines abolishes ALT-associated phenotypes [144,145], but ATRX, either by knocking out or knocking down in telomerase-positive cell lines did not present similar findings [138,145,146,147,148], suggesting that an ATRX loss alone is not sufficient for ALT activation. DAXX was originally identified as a Fas death receptor binding protein that induced apoptosis via JNK pathway activation. Thus, it was coined the death-domain-associated protein, DAXX [149]. ATRX and DAXX were initially seemed to be irrelevant. Analyzing H3.3 chaperone complexes identified ATRX and DAXX [150,151,152]. ATRX, in collaboration with DAXX, deposits H3.3 into telomeric and pericentromeric chromatin to prevent the formation of G-quadruplex DNA (G-4 DNA), a type of DNA structure that promotes homologous recombination repair and DNA-repair mechanisms, leading to telomere shortening [127,144,153]. These results reinforce the value of the ATRX/DAXX/H3.3 complex in ALT suppression (Figure 3). Gliomas with a WT *TERT* promoter frequently harbor mutations of *ATRX* to activate *ALT* [44]. Yang et al. successfully switched the telomere lengthening machinery of telomerase-positive cancer cells (HTC75) to an ALT-mediated telomere elongation mechanism by knocking out the TERT, inducing telomeric DNA damage, and disrupting the ATRX/DAXX complex [154], suggesting that ATRX and TERT mutations are mutually exclusive in conferring a selective growth advantage in cancers through telomere maintenance. Therefore, effective treatments should not only target TA in cancer cells but should also be aimed atmodulatingthe proper function of the ATRX/DAXX/H3.3 complex to destroy tumor cells.

## 5. Novel and Alternative Therapeutic Options for Treating GBM

There are several promising novel anticancer compounds with good potential for clinical applications. For example, the inhibition of mutant IDH may potentially have anticancer effects. Accordingly, the Food and Drug Administration (FDA) has approved the mutant IDH1 inhibitor, ivosidenib [155], and IDH2 inhibitor, enasidenib [156] for adults with relapsed or refractory acute myeloid leukemia, inhibitors of mutant IDH have entered clinical trials for glioma treatment [157,158]. Two compounds, precatorine and abrine, are reported to have promising effects against tumors with mutant IDH2/R140 [159]. Multiple approaches have been used to target telomerase through the development of vaccines, antisense oligonucleotides, and small-molecule inhibitors targeting TERT or TERC, such as BIBR1532, a small-molecule telomerase inhibitor, which disturbs the DNA substrate elongation upon template copying by reducing the number of TTAGGG repeats [160]; GRN163L, a direct telomerase RNA template antagonist binding with high specificity and affinity at the active site of TERT, causing a complete inhibition of the telomerase [161]; GV1001, an MHC II-peptide derived from the TERT vaccine with anti-inflammatory, anti-apoptotic, and antioxidant properties [162]; GRNVAC1, an autologous dendritic cell vaccine, consisting of RNA for the protein component of TERT and a portion of a lysosomal targeting signal, which guides the TERT to a lysosome enhancing its function to degrade into small peptides and leading to a stronger polyclonal immune response specific to all TERT epitopes expressed by patient tumors [163]; Vx-001, a TERT-derived peptide composed of a native cryptic peptide (TERT572) and an optimized variant (TERT572Y), showing significant inhibition of various types of tumor growth [164]; BRACO19, a promising small-molecule G-4 DNA stabilizing ligand, causing the shortening of telomeres [165]; geldanamycin, an HSP90 inhibitor, blocking the ATP-dependent binding of HSP90 to p23, causing disruption of the chaperone assembly leading to inhibition of the telomerase [166]; curcumin, a natural compound, causing time- and dose-dependent inhibition of the nuclear localization of TERT [167]; telomestatin, a natural product composed of a large array of polyoxazole rings that form macrocyclic linkages (R-telomestatin), leading to telomere shortening and apoptosis by activation of p38 MAPK, caspase-3, and poly-(ADP-ribose) polymerase [168]; and telomelysin, an oncolytic adenovirus with a modified gene containing a TERT promoter, showing promising antitumor activity through apoptotic and non-apoptotic pathways [169]. Although imetelstat, an oligonucleotide inhibitor, blocking the template region of telomerase [170], has been shown to inhibit TA and cancer cell proliferation in vitro and in animal [163] and in phase 1 studies [161], the regimen has proved too toxic in children with recurrent CNS tumors [171]. INO-5401, a combination of three DNA plasmids targeting the Wilms tumor gene-1 (WT1) antigen, the prostate-specific membrane antigen, and TERT genes, is being administered intramuscularly followed by electroporation in combination with cemiplimab, chemoradiation, and radiation to newly diagnosed GBM patients (NCT03491683). Meanwhile, eribulinmesylate, an FDA-approved drug for breast cancer and liposarcoma, has been shown to effectively and specifically inhibit the RNA-dependent RNA polymerase activity of TERT in multiple human GBM cell lines in preclinical mouse brain tumor models. Intraperitoneally administered erbulin also significantly prolongs the survival of mouse GBM models. Following these positive preclinical results, a phase 2 clinical trial is currently underway to investigate the use of eribulinmesylate in patients with recurrent GBM following the failure of treatment with temozolomide and bevacizumab [172].

Currently, the investigated agents targeting telomerase for cancer treatments are considered ineffective in ALT-dependent cancers or cancers adapting ALT mechanisms. Hence, to achieve effective tumor cell senescence and apoptosis by blocking telomere lengthening, not only should the telomerase-associated pathway be targeted, but the proper functioning of the ATRX/DAXX/H3.3 complex should be retained. Given the fast-paced development of genome editing technology, gene therapies targeting ATRX are considered to be among the potential treatments for ALT-positive cancers [154]. Additionally, as the association of *H3F3A* mutation with loss of ATRX and ALTis reported [128], and mutations in ATRX are found to cause changes in the patterns of DNA methylation of several highly repeated sequences [173], and DNA methylation patterns are tightly connected to histone 3 lysine K27 trimethylation (H3K27me3) patterns, and loss of H3K4me3 and retention of H3K4me2 or H3K27me3 are correlated with an increase in DNA methylation [54], these findings suggest that epigenic therapeutics targeting GBM by histone deacetylase (HDAC) inhibitors, including AR-42 [174], panobinostat [175], cyclic benzamides, hydroxamate derivates and Diallyl-trisulfides [176], andH3-K27M inhibitor, GSK-J4 [177] may be effective. Although several modern strategies to fight GBM through modulating telomerase or ALT are promising, there is still a long way to go to develop a complete cure for GBM.

Natural products have a long history of wide use as cancer treatments in China. Traditional Chinese medicines typically make use of plants or their derivatives and are usually considered alternative therapeutic options or palliative treatments, especially in the context of cancer treatment. However, there is an increasing amount of evidence supporting the use of plant extracts in direct and primary cancer treatments. For example, *Angelica sinensis* (Oliv.) Diels (AS), commonly known as dong quai, is used for the treatment of gastric mucosal damage, hepatic injury, chronic glomerulonephritis, impaired myocardial blood flow, and menopause in traditional Chinese medicine [178], and has been shown to have promising effects against brain cancers and other types of cancer [179,180,181,182,183]. The chloroform extract of *AS*, butylidenephthalide (BP), inhibits the proliferation of human GBM through the down-regulation of TERT and the consequent reduction in TA, leading to tumor senescence [184]. Meanwhile, BP has been found to show strong anti-proliferative effects against GBM in vitro and in vivo through inducing cell growth arrest and apoptosis [185]. In research involving two kinds of brain cancer cell lines, DBTRG-05MG and GBM 8401, BP was found to reduce TERT mRNA and protein expression in a dose-dependent manner, which is associated with the reduction of TA but independent of c-Myc regulation. By repressing TERT transcriptional activity, BP inhibits TA, an effect that is followed by human brain tumor cell senescence, such that the cells stay viable but stop synthesizing DNA and instead generate senescence-associated *β*-galactosidase [184]. In DBTRG-05MG tumor cells, BP promotes the phosphorylation of p53 and increases p53 expression; another two senescence-associated markers, p21 and p16, also increase after BP treatment. The BP-induced cell cycle arrest is associated with up-regulated cyclin-dependent kinase inhibitors (e.g., p21), which in turn, decreases the phosphorylation of retinoblasma proteins. Moreover, in BP-treated GBM cells, the level of apoptosis-associated proteins, such as caspase 8, procaspase 9, and procaspase 3, dramatically increases, and these proteins stay activated. Both p53-dependent and p53-independent pathways are shown to be involved in BP-induced apoptosis. In vivo, BP induces the shrinkage of in situ GBM and significantly prolongs survival [182]. Brain tumors are especially difficult to treat due to the BBB, which, by its nature, prevents toxins, large molecules, and xenobioticsin circulation from entering the brain. To overcome this barrier, the group involved in this study developed biodegradable polyanhydride wafers, which were tested by implanting them into xenograft animal models together with human GBM cells. The system served as an intracranial drug delivery instrument that enabled sufficient BP to reach the tumor site without causing significant harm to the surrounding normal brain tissue. The group found that the BP wafers dose-dependently reduced the size of brain tumors without relevant adverse effects in the animals. One of the critical mechanisms of these BP-wafers was the reduction in TERT mRNA expression, which leads to tumor senescence [186]. In sum, several studies reviewed herein demonstrate the substantial effects of BP against GBM, which supports further clinical investigations of the compound.

## 6. Conclusions

GBM can grow in all cortical areas, the brainstem, and the spinal cord. Patients with GBM may rapidly present with various signs and symptoms. It is so aggressive and invasive that even with maximal surgical resection plus radiotherapy with concomitant or subsequent chemotherapy, GBM remains an incurable tumor and a median survival of less than 2 years from diagnosis [12,15]. This review summarizes examples of current advances in the molecular mechanisms underlying GBM, such as mutant IDH, co-deletion at chromosome regions 1p/19q, mutations of H3F3A, telomerase, and ALT, and explores the potential of several novel and alternative therapeutic options, including various mutant IDH inhibitors [157,158]; telomerase vaccines, antisense oligonucleotides, peptide and small-molecule inhibitors targeting telomerase and TERT-targeting therapies [160,161,162,163,164,165,166,167,168,169]; gene therapies targeting ATRX [154]; epigenic therapeutics, such as histone deacetylase (HDAC) inhibitors [174,175,176] and methylation inhibitors [177]. In addition, plant-derived compounds, for example, BP, the chloroform extract of AS, has been found to inhibit the proliferation of GBM through the down-regulation of TERT and the consequent reduction in TA, leading to tumor apoptosis and senescence in vitro and in vivo [184,185]. However, GBM may use either one, or some, or all these mechanisms or another to cause excessive growth and progression, therefore, single-agent therapies may have no or low significant benefits. Moreover, because the crosstalk in signaling networks of these targets may affect the efficacy of the novel agents, effective treatments should not only target TA in cancer cells but should also be aimed at modulating the proper function of the ATRX/DAXX/H3.3 complex to destroy tumor cells. These therapies work in conjunction with the current standard of care, it is likely that patient survival and quality of life will be greatly improved with several modern strategies to fight GBM through modulating telomerase, epigenetics, or ALT. Of course, a wide range of additional investigations will be required to follow up on the promising leads generated by the studies reviewed herein, but taken together, these studies suggest various pathways to the more effective treatment of GBM and other gliomas in the future.
